# A systematic review of UK‐based long‐term nonsurgical interventions for people with severe obesity (BMI ≥35 kg m^−2^)

**DOI:** 10.1111/jhn.12732

**Published:** 2020-02-06

**Authors:** M. Aceves‐Martins, C. Robertson, D. Cooper, A. Avenell, F. Stewart, P. Aveyard, M. de Bruin, the REBALANCE team

**Affiliations:** ^1^ Health Services Research Unit (HSRU) University of Aberdeen Aberdeen UK; ^2^ Nuffield Department of Primary Care Health Sciences University of Oxford Oxford UK; ^3^ IQ Healthcare Radboud University Medical Centre Nijmegen The Netherlands

**Keywords:** severe obesity, UK, weight management programmes, BMI ≥35 kg m^−2^

## Abstract

**Introduction:**

The aim of this project was to systematically review UK evidence on the effectiveness of long‐term (≥12 months) weight management services (WMSs) for weight loss and weight maintenance for adults (≥16 years) with severe obesity (body mass index ≥35 kg m^−2^), who would generally be eligible for Tier 3 services.

**Methods:**

Four data sources were searched from 1999 to October 2018.

**Results:**

Our searches identified 20 studies, mostly noncomparative studies: 10 primary care interventions, nine in secondary care specialist weight management clinics and one commercial setting intervention. A programme including a phase of low energy formula diet (810–833 kcal day^−1^) showed the largest mean (SD) weight change at 12 months of –12.4 (11.4) kg for complete cases, with 25.3% dropout. Limitations or differences in evaluation and reporting (particularly for denominators), unclear dropout rates, and differences between participant groups in terms of comorbidities and psychological characteristics, made comparisons between WMSs and inferences challenging.

**Conclusions:**

There is a persistent and clear need for guidance on long‐term weight data collection and reporting methods to allow comparisons across studies and services for participants with severe obesity. Data could also include quality of life, clinical outcomes, adverse events, costs and economic outcomes. A randomised trial comparison of National Health Service Tier 3 services with commercial WMSs would be of value.

## Graphical abstract

This work systematically review UK evidence on the effectiveness of long‐term (≥12 months) weight management services for weight loss and weight maintenance for adults (≥16 years) with severe obesity (body mass index ≥35 kg m^−2^), who would generally be eligible for Tier 3 services in the National Health Service.

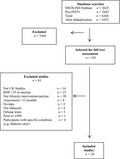

## Introduction

In the UK, obesity is managed on a tiered path by National Health Service (NHS) and community services. Tier 1 includes universal prevention services, Tier 2 includes lifestyle interventions in primary care, Tier 3 includes specialist multidisciplinary weight management services (WMSs) and Tier 4 includes bariatric surgery 1, 2, 3. Although people with severe obesity are likely to attend Tier 2 WMSs, having severe obesity (with or without comorbidities), may be a referral criterion for Tier 3 WMSs, prior to Tier 4 services 4, 5. Although adults with severe obesity may require more support with weight management, current National Institute for Health and Care Excellence (NICE) and Scottish Intercollegiate Guidelines Network (SIGN) guidance on WMSs provides little additional information for this group, apart from very‐low‐energy formula diets (VLEDs) (providing ≤800 kcal day^−1^) for people who need to lose weight quickly (e.g. for joint replacement or fertility treatment) 3, 4, 5, 6, 7, 8, 9. VLEDs are rarely used in the NHS, although there is increasing interest in the use of low energy formula diets (LEDs) (800–1200 kcal day^−1^). Prior attendance at a Tier 2 service may be a criterion for entering a Tier 3 service.

Effective services could reduce the numbers of patients moving on to higher tiers of weight management or contribute to the long‐term effectiveness after bariatric surgery. Our aim was to systematically review the UK evidence base for long‐term (≥12 months) behavioural interventions for weight loss and weight maintenance for adults with severe obesity [body mass index (BMI) ≥35 kg m^−2^] and evaluate their effectiveness.

## Materials and methods

The present study comprises an analysis of WMSs that are Tier 3 services or similar to Tier 3 services (e.g. participants with a spread of obesity‐related comorbidities and/or BMI ≥35 kg m^−2^) and is an updated version and a subgroup of results from the National Institute for Health Research funded *REview of Behaviour And Lifestyle interventions for severe obesity: AN evidenCE synthesis* (REBALANCE) 10 project. A protocol was registered *a priori* (PROSPERO No CRD42016040190). This systematic review is reported following the PRISMA standard 11.

### Inclusion criteria

Full‐text reports of UK WMSs of any study design published since 1999, in NHS clinical settings (e.g. primary care, secondary care) or commercial organisations, with a mean or median duration of ≥12 months of follow‐up, which included adults (mean or median age ≥16 years) with a mean or median BMI ≥35 kg m^−2^, were included. Studies focusing on participants with only one type of morbidity, as indicated by study inclusion and exclusion criteria, were excluded to reflect generalisable interventions for people with obesity and a range of comorbidities, rather than condition‐specific interventions, which would also have a behaviour change focus tailored for specific diseases, such as type 2 diabetes and weight management and blood sugar monitoring. Weight loss or prevention of weight regain after weight loss interventions (including VLEDs and LEDs), other dietary treatment, physical activity, behavioural counselling or a combination of these interventions were included. Interventions that included a pharmacological component (e.g. orlistat) were included only if this was offered as part of a WMS (i.e. studies were excluded for which the purpose was to evaluate orlistat).

The primary outcome was weight change or BMI change. Changes in secondary outcomes (e.g. cardiovascular risk factors) can be found in the full REBALANCE report 10.

### Literature searching

Literature searches were undertaken in four databases (MEDLINE, EMBASE, PsycINFO and Clinical Trials.gov) for interventions from 1999 to October 2018 10, 12, 13. ClinicalTrials.gov was searched for ongoing studies and reference lists of included studies were scanned to identify additional potentially relevant studies. Nineteen relevant NHS and commercial organisations, including Dietitians in Obesity Management, and the REBALANCE advisory group were contacted to help identify further published and unpublished reports. See REBALANCE report 10 for full search strategies.

The first, second and last author of the main included publications were contacted to identify additional materials (e.g. protocols, trial materials) that would assist data extraction.

### Data extraction and quality assessment

Three reviewers (MA‐M, CR and FS) independently screened titles, abstracts and full text reports, with a 10% check for agreement. The Template for Intervention Description and Replication (TIDieR) checklist was used for data extraction 14. Each reviewer extracted details of study design, methods, participants, interventions and outcomes, and TIDieR 14. A second reviewer (AA) checked numerical data extraction. Data for weight change are presented for complete cases, imputed estimations, last observation carried forward or baseline observation carried forward, as presented by authors.

Three reviewers (MA‐M, CR and FS) conducted a double‐blinded quality assessment of the included studies. The Cochrane risk of bias tool was used to assess randomised controlled trials (RCTs) 15 and a 17‐question quality assessment tool (ReBIP) was used to assess nonrandomised comparative and case series studies 16. An adapted version of the Campbell and Cochrane Equity Methods Group checklist 17 was used to assess the effect of interventions on disadvantaged groups and/or their impact on reducing socio‐economic inequalities.

## Results

**Figure 1:**
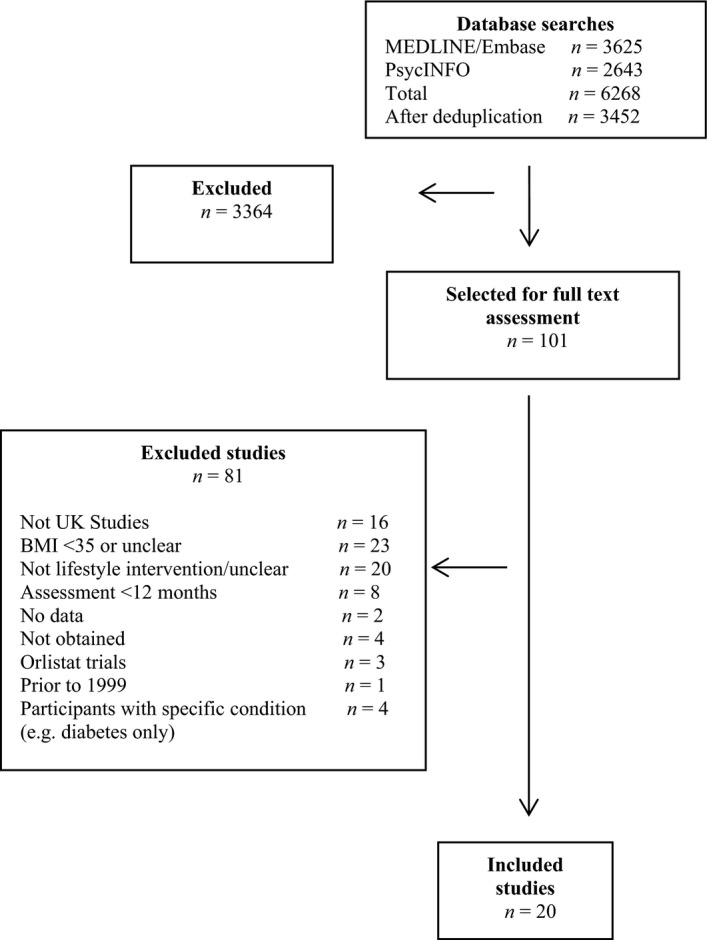
PRISMA diagram. BMI, body mass index.

Our searches identified 4078 potentially relevant titles and abstracts. From these, 20 18, 19, 20, 21, 22, 23, 24, 25, 26, 27, 28, 29, 30, 31, 32, 33, 34, 35, 36, 37, 38 studies were included (Fig. 1). Four were RCTs 18, 26, 29, 33, one 34 was a 9‐month RCT after a 3‐month nonrandomised screening period and the remaining 15 were observational studies.

General characteristics of the included studies are provided in Appendix 1. Ten WMSs were delivered in NHS primary care settings 18, 21, 22, 23, 25, 26, 28, 29, 31, 32. Nine were secondary care interventions at specialist weight management clinics 19, 20, 24, 27, 30, 33, 35, 37 and one was a commercial setting intervention 34. Some 65% of the studies took place in England, 25% in Scotland and 10% in more than one country of the UK.

### Characteristics of the participants

In total, 22 406 participants started interventions and 8982 were included in the analyses at final follow‐up, although numbers were sometimes unclearly reported. Two studies included only women 30, 31. Sample size varied from 84 31 to 6715 22 participants. Women represented 76.1% of the total population. The average participant age (weighted mean) was 48.4 years. The youngest reported mean age was 39.9 years 33 and the oldest was 55.8 years 23. The average BMI (weighted mean) of all participants was 39.9 kg m^−2^, the lowest 31 reported mean BMI was 35 kg m^−2^ and the highest^37^ was 50 kg m^−2^. Of note, 8.2% of women included in the study by Cartwright 20 had a BMI ≥60 kg m^−2^.

Three studies 21, 22, 23 did not report exclusion criteria. One trial 18 and one study 32 excluded participants using pharmacological treatment for obesity (e.g. orlistat), whereas three others offered orlistat as an optional drug treatment within the intervention.^25^, ^28^, ^29^ One of the primary care trials excluded participants with a BMI ≥45 kg m^−2^ 29 and one trial excluded participants with a perceived incapability of walking 100 m 26. One trial 26 and one study 31 reported excluding participants with psychiatric conditions (including eating disorders).

Although the main shared participant characteristic of the included reports was a mean BMI ≥35 kg m^−2^, participants varied in terms of obesity‐related comorbidities. For example, the prevalence of type 2 diabetes among participants was reported by 12 studies; 18, 22, 24, 26, 28, 29, 32, 36, 37 ranging from 9% 29 to 34.4% 28. Other reported comorbidities were hypertension 18, 21, 24, 32, 36, 37, impaired fasting glucose 21, 23, 24, cardiovascular disease 20, 21, 29, 36, 37 and dyslipidaemia 19, 21, 36. Other comorbidities reported were arthritis 20, 37, joint pain 36, 37, sleep apnoea 20, 24, 36, depression 24, 36 and asthma 37. Some studies 25, 27, 31, 33, 35 did not report any comorbidity.

### Assessment of risk of bias

**Figure 2:**
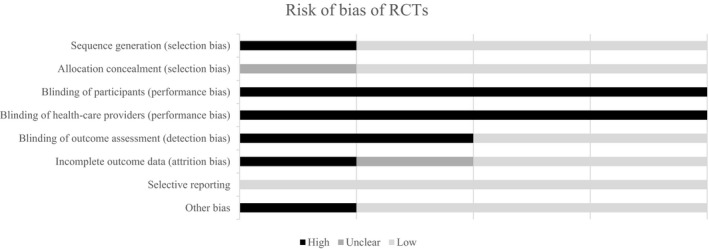
Risk of bias of randomised controlled trials (RCTs).

**Figure 3:**
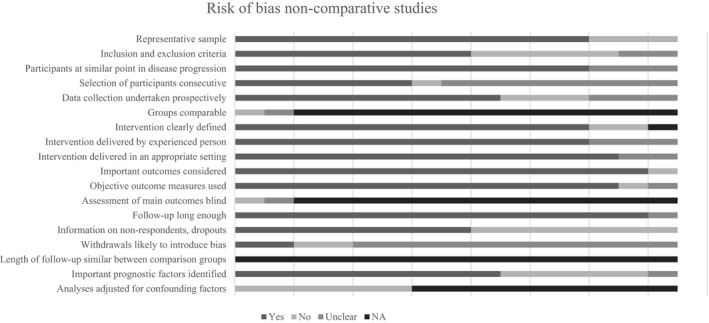
Risk of bias of nonrandomised comparative studies and case series.

The overall methodological quality was poor across studies (Figs 2 and 3). In four RCTs^18^, ^26^, ^29^, ^31^, many of the domains were assessed as being at a high risk of bias (Fig. 2). Only just over half of these studies (52.6%) provided information on participant dropouts.

### Assessment of equity and sustainability

**Figure 4:**
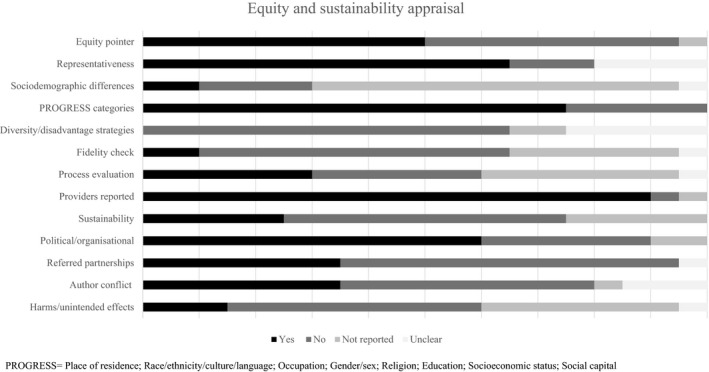
Equity and sustainability appraisal.

Half (50%) of the studies were conducted in settings that might target or exclude specific populations. Most (65%) did not report socio‐demographic differences between completers and withdrawals/dropouts, although 75%, reported details for some PROGRESS categories (Place of residence, Race/ethnicity, Occupation, Gender, Religion, Education, Socio‐economic status, or Social capital). Few (25%) considered sustainability, although 60% discussed their interventions in organisational contexts. Five studies 22, 25, 30, 32, 36 reported organisational partnerships (e.g. NHS, commercial organisations, local authorities and community groups) (Fig. 4).

Few studies assessed the fidelity of intervention delivery or participant adherence to interventions, and few reported intervention‐related adverse events. Potential for conflict of interest was unclear in 15% of studies.

One trial (Cambridge Weight Plan UK) 18 and one study (LighterLife Company) 25 received partial or full financial funding from the intervention manufacturer. In two further studies 24, 36, no conflict of interest was declared, but Cambridge Weight Plan UK donated products.

### Assessment of effectiveness

As a result of study heterogeneity, a narrative overview is presented according to the setting where the intervention was delivered.

### National Health Service primary care

Across primary care services, 10 eligible studies 18, 21, 22, 23, 25, 26, 28, 29, 31, 32 were identified. Most of these studies were undertaken in England, with the exception of two 21, 28 undertaken in different sites across the UK, as well as two 22, 25 in Scotland. In all cases, primary care practices were involved as the main setting of the studies, except one 28 that not only mainly recruited participants from primary care settings, but also included participants from commercial services (i.e. one commercial weight‐management service and recruitment through eight freelance Counterweight‐Plus trained practitioners). Women made up the majority of participants in primary care studies (over 60%) and one study recruited only women.^31^

The interventions were mainly delivered in primary care practices to individuals. One study also applied the intervention in pharmacies and community settings 22. The main care providers were nurses 21, 22, 23, 25, 26, 28, dietitians 21, 22, 25, 28, 31, 32, general practitioners or psychologists 29, 31. One trial described the intervention provider as a ‘LED counsellor’ 18. One study of primary care interventions incorporated other professionals, such as an exercise scientist 31. In most cases, the interventions were delivered individually, although three studies implemented group sessions 29, 31, 32.

One trial 18 and two 25, 28 studies evaluated the efficacy of LEDs in primary care, the latest in addition to the Counterweight programme 25, 28. In these three cases, the Cambridge Weight Plan/Counterweight PRO800 UK LED was offered (LED with 810–833 kcal day^−1^) and, in the study by Lean *et al*. 25, an option of an 810 kcal day^−1^ homemade LED was also available. Few of the interventions defined the nutritional characteristics of the dietary advice/or nutritional programme in depth 18, 21, 22, 25, 28, 31. Similarly, only one intervention provided in depth detail on the physical activity plan offered to participants 28.

General characteristics of the included studies delivered in the primary care studies are provided in Appendix 1. Overall weight, percentage of weight and BMI change are presented in Table 1.

**Table 1:** Weight change, percentage weight change and body mass index (BMI) change in National Health Service (NHS) primary care interventions

Study ID (first author, year, reference)	Intervention arm	Outcome measured	Baseline Outcome (SD) *n*	12‐Month Outcome, mean (SD) [% dropout]	24‐Month Outcome, mean (SD) [% dropout]
Astbury 2018 18 DROPLET Study	LED group 12 weeks of LED (810 kcal day^−1^) 4 weeks of food reintroduction	Weight (kg)	107.9 (18.9) *n* = 138	−10.2 (9.7)‡ −10.7 (9.6)† [24.6%]	–
		Weight change (%)	–	−9.5	–
		BMI (kg m^−2^)	37.6 (5.7)	−3.6	–
	Usual care group Appointments with a practice nurse	Weight (kg)	105.2 (20) *n* = 140	−3.5 (8.2)‡ −3.1 (7.0)† [32.1%]	–
		Weight change (%)	–	−3.3	–
		BMI (kg m^−2^)	36.8 (5.1)	−1.2	–
Jackson 2007 23	Specialist health visitor‐led, nonpharmacological intervention	Weight (kg)	103.2 (16.9) *n* = 89	−11.6 [Unclear]†	–
		Weight change (%)	‐	−11.2	–
		BMI (kg m^−2^)	37.4 (5.9)	−4.3	–
Lean 2013 25 Counterweight + LED	LED 12 weeks of LED (810–833 kcal day^−1^) 6–8 weeks of food reintroduction 34 weeks of weight maintenance	Weight (kg)	131.1 (25.2) *n* = 91	−12.4 (11.4) [25.3%]†	–
		Weight change (%)	–	−9.1 (8.2)	–
		BMI (kg m^−2^)	48 (7.6)	−4.5	–
Little 2017 26 POWeR+ Programme	(POWeR + face‐to‐face) 24 web‐based sessions designed to be used over 6 months plus nurse support	Weight (kg)	102.4 (16.9) *n* = 269	−3.8 [17.8%]†	–
		Weight change (%)	–	−3.7	–
		BMI (kg m^−2^)	36.7 (5.4)	−1.4	–
	(POWeR+Remote group) 24 web‐based sessions designed to be used over 6 months	Weight (kg)	102.9 (18.3) *n* = 270	−3.2 [19.3%]†	–
		Weight change (%)	–	−3.1	–
		BMI (kg m^−2^)	36.3 (5.7)	NR	–
	Nurse follow‐up Simple advice and simple materials to support behaviour change	Weight (kg)	104.4 (21.1) *n* = 279	−2.8 [18.6%]†	–
		Weight change (%)	–	−2.5	–
		BMI (kg m^−2^)	37.1 (6)	NR	–
McRobbie 2016 29 The WAP Programme	WAP Group‐based weight loss programme over eight weekly sessions followed by 10 monthly maintenance sessions	Weight (kg)	95.5 (15.8) *n* = 221	−4.2 (7.3) [32.5%]‡ −4.2 (7.3)†	–
		Weight change (%)	–	−4.4	–
		BMI (kg m^−2^)	35.0 (4.2)	−1.5 (2.6)	–
	Nurse follow‐up Best‐practice intervention incorporating national guidelines and NHS materials	Weight (kg)	98.3 (16.6) *n* = 109	−2.3 (6.6) [23.8%]‡ −2.3 (6.6)†	–
		Weight change (%)	–	−2.3	–
		BMI (kg m^−2^)	35.7 (4.3)	−0.8 (2.3)	–
Read 2004 32	Intervention Seven 2‐hour education and support group sessions to improve lifestyles	Weight (kg)	108 (20) *n* = 216	−11.5 [66.2%]†	–
		Weight change (%)	–	−10.6	–
		BMI (kg m^−2^)	39.7 (6.9)	−4.2	–
Ross 2008 21 Counterweight Programme (UK)	Intervention Trained general practice staff to deliver patient education and the transfer behaviour change skills	Weight (kg)	101.1 (NR) *n* = 1906	−3.0 (6.6) [54.8%]†	−2.3 (8.7) [56.7%]
		Weight change (%)	–	−2.9	−2.3
		BMI (kg m^−2^)	37.1 (6.0)	−1.1 (2.4)	NR
Ross 2012^22^ Counterweight Programme (Scotland)	Intervention Trained general practice staff to deliver patient education and the transfer behaviour change skills	Weight (kg)	NR *n* = 6715	−3.7 (12.2) [72%]†	–
		Weight change (%)	–	NR	–
		BMI (kg m^−2^)	37.0 (6.2)	NR	–
McCombie 2019 28 Counterweight + LED	Intervention 12 weeks of LED (853 kcal day^−1^) 12 weeks of food reintroduction Weight maintenance follow‐up until 12 months	Weight (kg)	128.0 (32.0) *n* = 288	−14.2 (11.6) [44.2%]† −10.5 (9.5) imputed −10.9 (11.6)‡ LOCF −7.9 (11.1)‡ BOCF	−13.5 (14.8)† [Unclear]
		Weight change (%)	–	−11.1	NR
		BMI (kg m^−2^)	45.7 (10.1)	−5.1	NR
Rapoport 2000 31	Modified version of cognitive behavioural therapy Cognitive principles incorporating incorporated elements from psychoeducational, nondieting and feminist approaches over a 10‐week period in group sessions	Weight (kg)	94.0 (16.1) *n* = 37	−1.9 [18.9%]†	‐
		Weight change (%)	–	−2	–
		BMI (kg m^−2^)	35.4 (6.3)	−0.9	–
	Standard cognitive behavioural therapy	Weight (kg)	94.8 (16.3) *n* = 38	−3.3 [26.3%]†	–
		Weight change (%)	–	3.4	–
		BMI (kg m^−2^)	35.3 (5.6)	−1.1	–

BOCF, baseline observation carried forward; LED, low‐energy formula diet (800–1200 kcal day^−1^); LOCF, last observation carried forward; m, meters; NR, not reported; VLED, very‐low‐energy formula diet (<800 kcal day^−1^).

^†^ Complete cases.

^‡^ Analysis adjusted for missing data.

In primary care, studies that provided LEDs were those with the higher weight loss. For example, after 12 months of follow‐up, Lean *et al*. 25 reported a mean (SD) weight loss of 12.4 (11.4) kg for completers with 25.3% drop out from baseline. A similar result was reported by Astbury *et al*. 18 where those participants randomised to LED were reported to have a mean (SD) weight loss of 10.7 (9.6) kg for completers [10.2 (9.7) kg by multiple imputation] and a dropout rate of 24.6%. In another study incorporating a LED, McCombie *et al*. 28 reported a mean (SD) weight loss of 14.2 (11.6) kg at 12 months for complete cases [−10.5 (9.5) kg imputed data] with a dropout rate of 44.2%.

From those interventions that did not include VLEDs or LEDs, higher reported weight losses were associated with higher dropout rates, which reflected selective reporting of results. Most primary care studies that did not include VLEDs or LEDs achieved weight losses at 12 months of 2–4 kg mostly for complete cases and dropout rates of 20–30%, with the exception of Counterweight studies where dropout rates were 55–72% at 12 months.

### Secondary care (specialist weight management clinics)

Nine studies evaluated specialist weight management clinics in the UK 19, 20, 21, 24, 27, 30, 35, 36, 37. Seven of these services were delivered in England and two were in Scotland 27, 33. Only one study was conducted as a RCT 33.

All WMSs included multidisciplinary teams (mainly a physician with a special interest in obesity, dietitians, and psychologists) and offered a similar service (behavioural therapy, including reduced calorie diets, LEDs, VLEDs and, in some cases, orlistat). Some interventions were delivered as individual sessions 20, 27, 33, 37, two were delivered as group sessions 19, 30, and three were delivered as both individual and group sessions 24, 35, 36. Some of the interventions were delivered in general practitioner practices in the community 20 or in local gyms 24. Only four studies provided weight data after 12 months of follow‐up 19, 20, 24, 37. Dropout rates, where clearly provided, ranged from 45% 24 to 78.3% 35 over the first 12 months.

Some interventions included an initial period with LED, and a follow‐up period with psychological and dietetic support.^18^, ^25^, ^28^ The number of contacts followed a similar pattern: intensive initial care (approximately the first 3 months) and then fortnightly or monthly meetings, comprising five to 15 contacts in the first 12 months.

Overall weight, percentage of weight and BMI change are presented in Table 2.

**Table 2:** Overall weight change, percentage weight change and body mass index (BMI) change in specialist weight management clinics

Study ID (first author, year, reference)	Intervention arm	Outcome measured	Baseline Outcome, mean (SD) *n*	12‐month Outcome, mean (SD)[% dropout]	24‐month Outcome, mean (SD) [% dropout]	36‐month Outcome, mean (SD) [% dropout]
Barrett 1999 19	VLED (600–800 kcal day^−1^)	Weight (kg)	119.8 (23.2) *n* = 115	−13.4 (10.0) [Unclear]†	−7.8 (9.8) [Unclear]†	–
		Weight change (%)	–	−10.9 (NR)	−6.6 (NR)	–
		BMI (kg m^−2^)	43.9 (7.5)	−4.9	−2.8	–
Cartwright 2014 20	Individual multidisciplinary care	Weight (kg)	132.1 (24.7) *n* = 262	−7 (10.8) [67.9%]†	−10.5 (18.7) [88.2%]	−13.4 (15.2) [91.6%]
		Weight change (%)	–	−5 (8.0)	−7.2 (10.9)	−10.2 (11.8)
		BMI (kg m^−2^)	47 (7.9)	−2.6 (4.0)	−3.5 (5.6)	−4.8 (5.6)
Rolland 2009 33	Low fat, 600 kcal day^−1^ deficit diet	Weight (kg)	NR	−17.5 (6.4) [Unclear]†	–	–
		Weight change (%)	–	NR	–	–
		BMI (kg m^−2^)	NR	NR	–	–
	Low carbohydrate/high protein (800–1500 kcal day^−1^) diet	Weight (kg)	NR	−3.0 (6.7) [Unclear]†	–	–
		Weight change (%)	–	NR	–	–
		BMI (kg m^−2^)	NR	NR	–	–
	VLED (550 kcal day^−1^)	Weight (kg)	NR	−16.1 (19.0) [Unclear]†	–	–
		Weight change (%)	–	NR	–	–
		BMI (kg m^−2^)	NR	NR	–	–
Ryan 2017 35	Patients who attended a specialist weight management service	Weight (kg)	127.2 (23.0) *n* = 141	−6.5 (11.5) [Unclear] −6.2 (11.5)†	–	–
		Weight change (%)	–	5.1	–	–
		BMI (kg m^−2^)	46.3(7.2)	−2.4	–	–
Steele 2017 36	Personalised plan including dietetics, physiotherapy, and behavioural therapy	Weight (kg)	127.1 (23.3) *n* = 1929	−4.0 (8.6)† [Unclear] −1.3 (5.3) BOCF‡ −2.9 (7.6) LOCF‡	–	–
		Weight change (%)	–	–	–	–
		BMI (kg m^−2^)	45.6 (6.8)	NR	–	–
Jennings 2014 24	The Fakenham weight management service	Weight (kg)	124.4 (27.3) *n* = 230	−10.2 (8.1) [45%]†	−9.6 (12.8) [Unclear]	−5.9 (10.7) [*Unclear*]
		Weight change (%)	–	−8.0 (6.0)	−7.1 (9.0)	−5.1 (9.1)
		BMI (kg m^−2^)	44.1 (7.8)	−2.1	−1.7	−0.9
Logue 2014 27	Greater Glasgow and Clyde WMS	Weight (kg)	118.1(52.6–244.8 range) *n* = 1838	−1.6 (5.5) [78.3%] BOCF‡	–	–
				−3.6 LOCF‡		
		Weight change (%)	–	NR	–	–
		BMI (kg m^−2^)	43.3 (NR)	NR	–	–
Packianathan 2005 30	900 kcal day^−1^ plus dietetic and behavioural therapy	Weight (kg)	95.1 (13.2) *n* = 150	−5.1 [69.3%]†	–	–
		Weight change (%)	–	−5.3	–	–
		BMI (kg m^−2^)	36.1 (5.6)	−2.2	–	–
Wallace 2015 37 Live Life Better Programme	Intensive lifestyle modification‐based programme	Weight (kg)	139.4 (28.6) *n* = 489	−11.8 (7.3) [Unclear]†	−14.9(8.7) [Unclear]	−18.2 (8.7) [Unclear]
		Weight change (%)	–	−8.5	−10.7	−13
		BMI (kg m^−2^)	50 (7.9) *n* = 487	−4.2 (2.5)	−5.2 (2.6)	−6.2 (2.7)

BOCF, baseline observation carried forward; kg, kilogramme; LED, Low‐energy formula diet (800–1200 kcal day^−1^); LOCF, last observation carried forward; m, meter; NR, not reported; VLED, very‐low‐energy formula diet (<800 kcal day^−1^).

† Data for those who completed.

‡ Adjusted for dropouts.

Data relate to 1249 who attended >2 sessions.

Rolland *et al*. 33 implemented a RCT. Patients initially underwent a dietary treatment with a low‐fat, 600 kcal day^−1^ deficit diet for 3 months. If patients responded well to this method, it was continued for the next 9 months. If patients failed to lose weight, they were randomised either to LighterLife VLED (550 kcal day^−1^) plus a weekly group support activity or a low carbohydrate/high protein (800–1500 kcal day^−1^) diet for the next 9 months with six contacts over 9 months. After 12 months, participants who responded well to the initial low fat, 600 kcal day^−1^ deficit diet (and were not randomised), had the highest weight change of all participants within this trial [−17.5 (6.4) kg] and across the other studies set in secondary care clinics, although the dropout rate was unclear for this group. 12‐month weight loss in the VLED group was 16.1 (19.0) kg compared to 3.0 (6.7) kg for the low carbohydrate high protein diet. Dropout rates were also unclear for these groups.

Across other studies that included a LED or VLED, weight loss varied from 5.1 kg 30 to 13.4 kg 19 after 12 months; however, the dropout rates were either unclear or over 69%.

### Commercial setting

Only one study was conducted outside the NHS setting. Rolland *et al*. 34 retrospectively assessed the effect of LighterLife Total VLED with group‐based behaviour therapy for self‐referred participants who completed 1 year of treatment. The initial weight loss phase could vary from weeks to several months, continued by weekly group meetings. The mean (SD) weight change from baseline was −12.9 (11.3) kg at 36 months, presumed for completers; dropout rates were unclear. Over 50% of participants returned to the weight loss phase for a second attempt during the 36‐month period (Table 3).

**Table 3:** Overall weight change, percentage weight change and body mass index (BMI) change in commercial setting, presumed data for completers

Study ID	Intervention arm	Outcome measured	Baseline outcome, mean (SD) *n*	12‐month Outcome, mean (SD) [% dropout]	24‐month Outcome, mean (SD) [% dropout]	36‐month Outcome, mean (SD) [% dropout]
Rolland 2014 34 LighterLife	VLED (550 kcal day^−1^)	Weight (kg)	99.1 (16.6) *n* = 5965	−18 (11.4) [Unclear]	−14.9 (11.4) [Unclear]	−12.9 (11.3) [Unclear]
		Weight change (%)	–	−17.6 (9.5)	−14.7 (10)	−12.9 (10)
		BMI (kg m^−2^)	36.3 (5.1)	−6.6	−5.4	−4.7

VLED, very‐low‐energy formula diet (<800 kcal day^−1^).

## Discussion

We attempted to comprehensively review studies relevant to Tier 3 WMSs for adults with higher BMIs. One previous systematic review of Tier 3 weight loss services for adults by Brown *et al*. 38 included 14 studies with wider BMIs and shorter follow‐up. Our focus was somewhat different, looking at longer‐term outcome data from services relevant to adults with a BMI ≥35 kg m^−2^. The distinction between Tier 2 and Tier 3 services appears to be blurred. Two specialist weight management services 27, 35 explained that participants needed to undertake a programme similar to Tier 2 services before entering their Tier 3 programme. Primary care services offered programmes to participants whose mean was BMI ≥35 kg m^−2^ with a range of comorbidities; these programmes were difficult to distinguish from those for participants in secondary care specialist weight management services in the studies reported here.

Only 35% of our included studies reported data beyond 12 months; the absence of long‐term data in the remaining studies is problematic with repect to evaluating the long‐term effectiveness of these interventions. Limitations or differences in evaluation and reporting, as well as differences between participant groups in terms of comorbidities and psychological characteristics, made comparisons and inferences between studies and interventions challenging, and precluded meta‐analysis. There is a need to improve data collection data in these interventions. Long‐term data collection has been a challenge, in terms of funders providing resources to allow this to happen.

Across studies, LEDs were associated with the greatest weight losses; for example, a mean weight change of –12.4 kg at 12 months in the study by Lean *et al*. 25, with a reported dropout rate of 25.3% 25, as well as similar results in the study by Astbury *et al* 18. Dropout rates tended to be lower with LEDs, which could suggest that better weight loss with these diets provided participants with more motivation to continue in the weight management programme. Unclear denominators in studies with the LighterLife VLED do not allow comparisons with other VLED 19, 33 studies. Only one trial 26 described expressly considering participants' choices or motivations for improving engagement with starting or continuing services. By contrast, one study 24 reported excluding participants ‘by their lack of motivation’. Motivation (or lack of it) is sometimes assessed before participants are included in services, and so it would be helpful for authors to be explicit about this assessment and the referral pathway. Changing dietary advice according to how the weight of participants responds to different dietary interventions also appears to be beneficial for weight loss 33.

Socio‐demographic characteristics were often not reported and few studies appeared to include hard to reach or disadvantaged groups (e.g. ethnic groups, people with disabilities, younger or older people) or participants with a BMI >40 kg m^−2^.

All studies included both men and women, except for two women‐only studies 30, 31. Overall, more women (76.1%) were recruited than men in the remaining studies. Evidence was insufficient to assess whether specific services for men or women would be more effective. One study, which was not included in this review, reported the results obtained in a community intervention delivered in football clubs to men with mean BMI of 35 kg m^−2^ 39. Exceptionally, this trial showed little evidence of weight regain by 12 months; weight loss 5.6 (8.1) kg, 11.0% dropout at 12 months. The results of this study indicate that WMSs that are tailored for men could be particularly effective. Few interventions reported considering ‘emergency plans’ or contact after the intervention, if needed.

Dietary and physical activity interventions were poorly described, making programme reproduction difficult. One study 19 and one trial 29 did report participants' weight loss history (including number of past weight loss attempts, methods used, average weight lost). Some studies excluded participants with eating disorders 31, 33, 34. In one trial, participants were able to choose their diets 26. Important features of the diets (e.g. availability; affordability; preferences; behavioural, social and economic costs for participants) were not described. These factors could impact on intervention effectiveness and adherence. Similarly, the extent to which diets were tailored may influence not only their success, but also their ease of delivery.

One study 24 and one trial 26 provided information on physical activity advice provided to participants; however, in most cases, details of physical activity advice were either poorly reported or not reported at all. One trial excluded participants with inability to walk more than 100 m 26. Others included participants with arthritis 20, 37 or joint pain 36, 37, factors to consider when recommending physical activity.

Scaling up interventions to reach more participants is important, particularly from an NHS perspective. Little *et al*. 26 showed that remote delivery produced much the same 12 month weight change compared to face‐to‐face delivery with a dropout rate of under 20% (mean −3.2 kg and −3.8 kg, respectively, for completers). This is comparable to the 12‐month weight loss in the Counterweight evaluations 21, 22, which had dropout rates of 54.8% to 72%, although these are smaller weight losses than those reported in UK RCTs of commercial WMSs in primary care, with dropout rates from 11% to 29.5% 38, 40. Similarly, given that primary care referral to a commercial provider for participants of mean BMI 34.6 kg m^−2^ (in a RCT excluded from our review) demonstrated a weight loss of 4.9% from 12 weeks of programme at 12 months (100% of participants) and 7.1% from 52 weeks of programme (data for all participants), the role of commercial providers for people with higher BMIs could be explored further 41. A comparison of Tier 3 services with commercial WMSs would be of value, considering the possible methodological challenges that this might comprise (particularly data collection and drop‐out rates). Long‐term UK data are urgently needed for participants with severe obesity (e.g. LighterLife, Cambridge Weight Plan, Weight Watchers, Slimming World, Counterweight Ltd) with weight outcomes taking account of dropouts. Randomised evaluations of comparisons of different approaches, including existing Tier 3 specialist WMSs, or allowance for the choice of reducing diet, would be valuable.

None of the included studies reported adapting the intervention to the needs of participants. Interventions appear to have been designed according to the resource availability or capability of the weight management system. For example, none of the studies reported attending participants out of the practice's regular attendance hours (e.g. evening or weekends), to facilitate participation.

There is a clear need for guidance on weight data collection and reporting to allow comparisons across studies and services. It was difficult to make comparisons between services, particularly when data were not provided for all participants (e.g. by last observation carried forward or baseline observation carried forward, which correct for differences in dropout rates). Services should be funded to collect data for longer than 1 year, preferably for 5 years. Public Health England has guidance for the evaluation of weight loss services 42 and a core outcome set has been developed in the UK using consensus methods, including advice on weight change data collection and statistical analysis 43, 44. Data should include quality of life, clinical outcomes, adverse events, costs and economic outcomes in a standard format. More detailed guidance on the content of reported WMSs would be very valuable, aiding with replication and evaluation.

In summary, our searches identified 20 studies, which were mostly noncomparative. A programme including a phase of low energy formula diet low energy diet showed the largest mean weight change at 12 months of −12.4 (11.4) kg with 25.3% dropout. Differences in evaluation and reporting (particularly for denominators), unclear dropout rates, and differences between participant groups in terms of comorbidities and psychological characteristics, make comparisons between different programmes very challenging. There is a persistent and clear need for guidance on long‐term weight data collection and reporting methods to allow comparisons across studies and services for participants with severe obesity.

## Transparency declaration

The lead author affirms that this manuscript is an honest, accurate and transparent account of the study being reported. The reporting of this work is compliant with PRISMA guidelines. The lead author affirms that no important aspects of the study have been omitted and that any discrepancies from the study as planned (protocol PROSPERO No CRD42016040190). This project is part of the National Institute for Health Research funded *REview of Behaviour And Lifestyle interventions for severe obesity: AN evidenCE synthesis* (REBALANCE) 10 project.

## The REBALANCE Team

REBALANCE Project management team: Elisabet Jacobsen (Health Economics Research Unit, University of Aberdeen, Aberdeen, UK), Dwayne Boyers (Health Economics Research Unit, University of Aberdeen, Aberdeen, UK), David Cooper (Health Services Research Unit, University of Aberdeen, Aberdeen, UK), Lise Retat (HealthLumen, London, UK), Paul Aveyard (Nuffield Department of Primary Care Health Sciences, Oxford University, Oxford, UK), Fiona Stewart (Health Services Research Unit, University of Aberdeen, Aberdeen, UK), Graeme MacLennan (Health Services Research Unit, University of Aberdeen, Aberdeen, UK), Laura Webber (HealthLumen, London, UK), Emily Corbould (UK Health Forum, Fleetbank House, Salisbury Square, London, UK), Benshuai Xu (UK Health Forum, Fleetbank House, Salisbury Square, London, UK), Abbygail Jaccard (UK Health Forum, Fleetbank House, Salisbury Square, London, UK), Bonnie Boyle (Health Services Research Unit, University of Aberdeen, Aberdeen, UK), Eilidh Duncan (Health Services Research Unit, University of Aberdeen, Aberdeen, UK), Michal Shimonovich (Health Services Research Unit, University of Aberdeen, Aberdeen, UK), Cynthia Fraser (Health Services Research Unit, University of Aberdeen, Aberdeen, UK), Lara Kemp (Health Services Research Unit, University of Aberdeen, Aberdeen, UK). REBALANCE Advisory Group for all their advice and support during this project: Margaret Watson, Lorna Van Lierop, Richard Clarke, Jennifer Logue, Laura Stewart, Richard Welbourn, Jamie Blackshaw, Su Sethi.

## Appendix 1

**Table:**

Study (first author, year, reference)	Study characteristics	Participant´s characteristics	Intervention characteristics
Primary care services			
Astbury 2018 18 Doctor Referral of Overweight People to Low Energy total diet replacement Treatment (DROPLET) Study	**Location**: Primary care practices in Oxfordshire, England **Design**: Pragmatic, two arm, parallel group, open label, individually randomised controlled study **Period of the study**: 2016–2017 **Recruitment**: Participants sourced from 10 practices **Number of participants allocated**: 278 (*intervention*: 138, *control*: 140)	**Inclusion criteria**: BMI ≥30 kg m^−2^and age ≥18 years **Exclusion criteria**: People who had received or were scheduled for bariatric surgery, in a weight management programme, or with contraindications to the dietary intervention **Baseline age, mean (SD)**: 37.2 (5.4) **Comorbidities at baseline**: 23% had hypertension and 15% had diabetes	**Delivered by**: *Intervention:* untrained ‘counsellors’ and clinicians. *Control:* nurses **Description**: *Intervention group*: 8 initial weeks with a LED (810 kcal day^−1^), followed by 4 weeks of food reintroduction. Regular behavioural support was offered. *Usual care*: Series of appointments for behavioural weight management advice for 12 weeks **Duration of active intervention**: 24 weeks **Length of follow‐up (months)**: 12
Jackson 2007 23	**Location**: A moderately deprived health centre from West Yorkshire, England **Design**: Prospective study **Period of the study**: 2003–2004 **Recruitment**: Participants were referred to the clinic by the family physicians, practice‐based nurses and health visitors **Number of participants allocated**: 89	**Inclusion criteria**: BMI >35 kg m^−2^ or BMI >30 kg m^−2^ with associated comorbidities **Exclusion criteria**: NR **Baseline age, mean (SD)**: 55.8 (13.8) **Comorbidities at baseline**: 13.5% had impaired fasting glycaemia	**Delivered by**: Public health nurse **Description**: The goal of the clinic was to deliver a specialist health visitor‐led, nonpharmacological intervention to adopt a healthier lifexmlstyle through healthy eating and increasing physical activity **Duration of active intervention**: Appointments within 3 weeks of the initial referral, then at two weekly intervals for 12 months. Contact after 12 months was negotiated, depending on need **Length of follow‐up (months)**: 12
Read 2004 32	**Location**: Three health centres in the north locality of Nottingham City Primary Care Trust, England **Design**: Prospective study **Period of the study**: 2000–2002 **Recruitment**: GPs and practice nurses could refer patients opportunistically or patients could refer themselves **Number of participants allocated**: 216	**Inclusion criteria**: 18–65 years old, BMI >30 kg m^−2^ with associated comorbidities **Exclusion criteria**: current use of obesity medication, insulin treatment of diabetes, pregnancy, and attendance at a hospital obesity clinic **Baseline age, mean (SD)**: 50.4 (12.4) **Comorbidities at baseline**: 57% had hypertension, 25% had diabetes, 10% had angina, 9% had previous myocardial infarction	**Delivered by**: Dietitian and nurse **Description**: Individual assessment appointment before commencing the group sessions. Seven 2‐hour education and support group sessions to improve lifestyles run by the dietitian at intervals of 2 weeks. Further 2‐hour sessions were delivered at 4, 6, 9, and 12 months, **Duration of active intervention**: 12 months **Length of follow‐up (months)**: 12
McRobbie 2016 29 The Weight Action Programme (WAP)	**Location**: Six GP surgeries from areas with high levels of social deprivation across London, England **Design**: Randomised controlled trial **Period of the study**: 2012–2015 **Recruitment**: Primarily recruited from the practices, and further advertising was made **Number of participants allocated**: 330 (*intervention*: 214, *control*: 116)	**Inclusion criteria**: Age ≥18 years and BMI of ≥30 kg m^−2^ or ≥ 28 kg m^−2^ with associated comorbidities **Exclusion criteria**: BMI of >45 kg m^−2^, had lost > 5% of weight in the previous 6 months, were pregnant, were taking psychiatric medications **Baseline age, mean (SD)**: *Intervention* 46.6 (15.0) *Control* 45.1 (14.2) **Comorbidities at baseline**: *Intervention* 10% had heart disease, 10% had diabetes *Control* 6% had heart disease, 8% had diabetes	**Delivered by**: *Intervention* health psychologists. *Control* GPs and practice nurses **Description**: *Intervention* group‐based weight loss programme (10–20 participants) delivered over eight weekly group sessions followed by 10 monthly maintenance sessions that combine standard cognitive behavioural interventions, dietary advice and self‐monitoring with group‐oriented interventions. *Control* Best practice intervention incorporating national guidelines and NHS materials in four one‐to‐one sessions delivered over 8 weeks. Orlistat was an option to participants in both groups **Duration of active intervention**: *Intervention* 12 months *Control* 8 weeks **Length of follow‐up (months)**: 12
Rapoport 2000 31	**Location**: GP surgeries or local health clinics (geographical location not specified), England (by authors affiliation) **Design**: Randomised controlled trial **Period of the study**: Prior to 2000 **Recruitment**: through letters to GP, posters in health centres and notices in the local media **Number of participants allocated**: 75 (*intervention [modified cognitive‐behavioural therapy]*: 37, *control [standard cognitive‐behavioural therapy]*: 38)	**Inclusion criteria**: Women aged 18–65 years and BMI of ≥28 kg m^−2^ **Exclusion criteria**: being involved in any other method of weight management, serious medical or psychiatric conditions (including eating disorders), insulin dependent diabetes, and pregnancy or lactation **Baseline age, mean (SD)**: *Intervention* 49 (10) *Control* 46 (12) **Comorbidities at baseline**: None reported	**Delivered by**: Registered dietitian and a health psychologist, a clinical psychologist and an exercise scientist **Description**: Both treatment programmes involved weekly, 2h sessions over a 10‐week period, with around 10 participants in each group. *Intervention:* The programme emphasised regular physical activity and healthy eating as means to improve overall health rather than focusing in weight loss using used basic behavioural and cognitive principles incorporating incorporated elements from psychoeducational, nondieting and feminist approaches. *Control:* Moderate energy deficit giving approximately 1200 kcal day^−1^. Participants were asked to set specific weight loss goals, basic behavioural and cognitive principles **Duration of active intervention**: 10 weeks **Length of follow‐up (months)**: 12
Little 2017 26 POWeR+ (Positive Online Weight Reduction) Programme	**Location**: General practices around the centres of Southampton and Oxford, England **Design**: Randomised parallel‐group study **Period of the study**: 2013–2014 **Recruitment**: General practices identified participants from their electronic records, and up to 100 patients from each practice were randomly chosen and invited by letter **Number of participants allocated**: 826 (*intervention [face‐to‐face]*: 269, *intervention [remote]*:270 *control*: 279)	**Inclusion criteria**: BMI of ≥30 kg m^−2^ or ≥ 28 kg m^−2^ with associated comorbidities **Exclusion criteria**: Major mental problems, very severe illness (difficulty completing outcomes and were unable to change diet), were pregnant or breastfeeding, or had a perceived inability to walk 100 m **Baseline age, mean (SD)**: *intervention [face‐to‐face]*: 53.7 (13.2), *intervention [remote]*: 54.7 (13) *control*: 52.7 (13.3) **Comorbidities at baseline**: 17% in the intervention [face‐to‐face], 16% in the intervention [remote] and 17% in the control group had diabetes	**Delivered by**: Nurses **Description**: *Control*: advice and simple materials to support behaviour change. *Intervention [face‐to‐face]*: Web intervention to teach patients self‐regulation and cognitive behavioural techniques to form sustainable eating and physical activity, 24 web‐based sessions designed to be used over 6 months. Participants had three scheduled face‐to‐face appointments in the first 3 months and then up to four more during the next 3 months. *Intervention [remote]*: Patients could access the same web‐based intervention as in the face‐to‐face group and the intervention was to assess whether even briefer professional support for the web intervention could be effective. In addition to 6 monthly weighing, as in the control group, participants had three scheduled telephone or e‐mail contacts and up to two optional telephone/e‐mail contacts during the first 6 months) **Duration of active intervention**: 6 months **Length of follow‐up (months)**: 12
Ross 2008 21 Counterweight Programme Project (UK)	**Location**: 65 general practices from seven UK regions **Design**: Prospective study **Period of the study**: 2000–2005 **Recruitment**: Patients were identified by GPs and practice nurses during normal appointments **Number of participants allocated**: 1906	**Inclusion criteria**: Age 18–75 years and a BMI of ≥30 kg m^−2^ or ≥ 28 kg m^−2^ with associated comorbidities **Exclusion criteria**: Not reported **Baseline age, mean (SD)**: 49.4 (13.5) **Comorbidities at baseline**: 13.5% had diabetes, 32.1% had hypertension, 12.5% had dyslipidaemia, 8% had cardiovascular disease and 9.9% had impaired glucose	**Delivered by**: Practice staff (GPs, nurses and healthcare assistants) trained by registered dietitians with expertise in obesity management **Description**: The practice nurse/healthcare assistant role was to deliver patient education through discussion about weight management, communication of information, and the transfer of behaviour change skills and strategies during weight management sessions. The aim was to achieve an energy deficit of 500–600 kcal day^−1^. Participants were asked to commit to nine appointments in 12 months (included six initial appointments of 10–30 minutes each, with follow‐up visits at 6, 9 and 12 months) **Duration of active intervention**: 12 months **Length of follow‐up (months)**: 24
Ross 2012 22 Counterweight Programme Project (Scotland)	**Location**: 13 Health Boards (including 184 general practices, 16 pharmacies), Scotland. Mainly delivered in general practices, but one Health Board chose a pharmacy setting and another favoured community‐based implementation of the programme **Design**: Prospective study **Period of the study**: 2006–2008 **Recruitment**: Counterweight Programme was positioned alongside ‘Keep Well’ for practice recruitment and screening of patients **Number of participants allocated**: 6715	**Inclusion criteria**: 40–64 years (specification for the ‘Keep Well’ programme), BMI of ≥30 kg m^−2^ or ≥ 28 kg m^−2^ with associated comorbidities **Exclusion criteria**: Not reported **Baseline age, mean (SD)**: 53.0 (10.4) **Comorbidities at baseline**: From those enrolled by 16 community pharmacies (*n* = 458), 11.6 % had diabetes	**Delivered by**: Practice staff (GPs, Nurses and healthcare assistants) trained by registered dietitians with expertise in obesity management **Description**: As described previously (see Ross *et al*. 21). **Duration of active intervention**: 12 months **Length of follow‐up (months)**: 12
Lean 2013 25 Feasibility study for Counterweight Plus programme	**Location**: Practices already delivering Counterweight, predominately in rural or small‐town settings in Scotland **Design**: Prospective study **Period of the study**: Prior to February 2013 **Recruitment**: Participants were proposed by GPs, practice nurses, or local dietitians **Number of participants allocated**: 91	**Inclusion criteria**: 20–60 years with BMI ≥40 kg m^−2^ **Exclusion criteria**: pregnancy or lactation, diabetes and taking insulin, myocardial infarction cancers, chronic pancreatitis, alcohol dependence, psychiatric illness, and learning disability **Baseline age, mean (SD)**: 45.7 (10.7) **Comorbidities at baseline**: Not reported	**Delivered by**: Practice nurses, physicians and dietitians **Description**: The intervention was delivered in practices that were delivering the Counterweight programme (see Ross *et al*. 22). There was an initial phase of 12 weeks of LED (810–833 kcal day^−1^) with weekly appointments for the first 12 weeks. Then a food reintroduction phase of 6–8 weeks with one 360–400 kcal meal day^–1^ followed by a weight maintenance phase of 34 weeks. All nutrition from food was based on individualised food portion plan based on 500–600 calorie deficit day^–1^ with an upper limit of 2500 kcal day^−1^ in the last phase. 30 min per day of moderate physical activity was encouraged. Telephone support was provided if necessary. Orlistat was optional for participants **Duration of active intervention**: 12 months **Length of follow‐up (months)**: 12
McCombie 2019 28 Counterweight‐Plus Programme Project (UK)	**Location**: A variety of UK providers **Design**: Prospective study **Period of the study**: 2013–2018 **Recruitment**: Participants recruited from nine UK Health Service areas, one private weight management service, eight private freelance Counterweight‐Plus trained practitioners **Number of participants allocated**: 288	**Inclusion criteria**: Age 18–75 years and a BMI of ≥30 kg m^−2^ or ≥ 28 kg m^−2^ with associated comorbidities **Exclusion criteria**: Active mental illness, myocardial infarction or stroke within the previous 3 months, severe or unstable heart failure, porphyria, pregnant and until >4 months post‐partum, breastfeeding, substance abuse or eating disorder accompanied by purging **Baseline age, mean (SD)**: 45.7 (12.7) **Comorbidities at baseline**: 34.4 % had diabetes (97% type 2 diabetes and 3% type 1 diabetes)	**Delivered by**: registered healthcare professionals (mainly registered dietitians) with specialist training in weight management, with access to consultant physician expertise **Description**: Seven 60 min appointments over 12 weeks (or up to 20 weeks if greater weight loss required), where LED (825–853 kcal day^−1^) products and written resources are provided. Then a food reintroduction phase with six appointments of 20 min over 6–12 weeks. Increased physical activity, 30 min of moderate activity day^–1^ at least 5 days/week. Once achieved, aim for 45–60 min of moderate activity day^–1^ (monitoring with step‐counters or activity trackers if possible). Orlistat available depending on local prescribing access. Seven appointments given to consolidate behavioural change strategies and restrict weight regain **Duration of active intervention**: 12 months **Length of follow‐up (months)**: 12
NHS Specialist Weight Management Clinics (Secondary Care)			
Barrett 1999 19	**Location**: The Luton and Dunstable Hospital specialist multidisciplinary obesity services, England **Design**: Retrospective study **Period of the study**: Prior to 1999 **Recruitment**: Patients referred by General Practitioners **Number of participants allocated**: 115	**Inclusion criteria**: Referral to the clinic was often prompted by physical health problems related to obesity **Exclusion criteria**: Lack of motivation or an eating disorder **Baseline age, mean (SD)**: 42 (NR) **Comorbidities at baseline**: 34% had hypertension; 11% had non‐insulin dependent diabetes and 41% had dyslipidaemia	**Delivered by**: Consultant physician, clinical psychologist and a senior dietitian **Description**: Seven closed group sessions providing formalised behaviour and cognitive modification combined with an initial VLED (600–800 kcal day^−1^). Pharmacology treatment was given upon evaluation. After completing 12‐week programme, patients returned to clinic at 3‐month intervals for advice and weighing. **Duration of active intervention**: 12 weeks **Length of follow‐up (months)**: 18
Cartwright 2014 20	**Location**: Specialist Weight Management Heart of England NHS Foundation Trust and the former South Birmingham Primary Care Trust (but the programme was delivered at local general practices), England **Design**: Prospective study **Period of the study**: 2008–2012 **Recruitment**: Patients referred from primary care settings in West Midlands **Number of participants allocated**: 262	**Inclusion criteria**: Age 19–76 years with BMI of ≥40 kg m^−2^ or ≥ 35 kg m^−2^ with associated comorbidities **Exclusion criteria**: Not reported **Baseline age, mean (SD)**: 43.1 (11.8) **Comorbidities at baseline**: 26.3% had diabetes, 11.1% had cardiovascular disease, 34.4% had hypertension, 24% had arthritis, and 25.6% had obstructive sleep apnoea	**Delivered by**: Physicians, dieticians and a psychologist **Description**: Comprehensive multidisciplinary care delivered through individual appointments at GP practices. The frequency of contact was every three months, but varied with individual requirements and session availability, with individuals attending subsequent appointments every two to three months or more frequently if needed. Totalling a range of contacts from 5 to 13 **Duration of active intervention**: 12 months **Length of follow‐up (months)**: 36
Rolland 2009 33	**Location**: Specialist Obesity Clinic, Scotland **Design**: Randomised controlled trial **Period of the study**: Prior to 2009 **Recruitment**: Patients were referred by primary care services **Number of participants allocated**: 120 (After three months: *VLED group* 34, *Low carbohydrate group* 38, *Energy deficient group* 18)	**Inclusion criteria**: Age over 18 years with BMI of ≥35 kg m^−2^ **Exclusion criteria**: history of hepatic or renal disease, cancer, currently pregnant or lactating, on antidepressants or anti‐obesity medication, eating disorders **Baseline age, mean (SD)**: Not available for the whole sample. *VLED* 39.9 (10.4), *Low‐carbohydrate group* 42.7 (13.1) **Comorbidities at baseline**: Not reported	**Delivered by**: Physician and dietitian **Description**: Patients initially underwent a dietary treatment with a low fat, 600 kcal day^−1^ deficit diet for three months. If patients responded well, it was continued for 9 months. If patients fail to lose weight with it, they were randomised to LighterLife VLED (550 kcal day^−1^) plus group support weekly or a low carbohydrate/high protein (800–1500 kcal day^−1^) diet for 9 months **Duration of active intervention**: 12 months **Length of follow‐up (months)**: 12
Packianathan 2005 30	**Location**: England, no other details **Design**: Longitudinal study **Period of the study**: Priori to July 2005 **Recruitment**: Through advertisements in local news media **Number of participants allocated**: 150	**Inclusion criteria**: Women, aged 35–65 years, with a BMI 35–45 **Exclusion criteria**: If women were dieting, had a secondary cause of obesity, were on drugs known to affect energy balance, had a history of eating disorder, had lactose intolerance or had significant comorbidity **Baseline age, mean (SD)**:48.5 (8.3) **Comorbidities at baseline**: Excluded if participants had a comorbidity	**Delivered by**: Dietitian and physicians **Description**: Phase 1 included a 16‐week acute weight loss intervention with 900 kcal day^−1^ with SlimFast plus biweekly for a one hour dietetic and cognitive behavioural therapy. Second phase up to 10 SlimFast meal replacements/week, optional 900 kcal day^−1^ for relapse, or patients could choose a low‐fat diet with a 600 kcal day^−1^ energy deficit, plus group dietetic and lifestyle therapy, behavioural modification and advice on increased physical activity **Duration of active intervention**: 12 months **Length of follow‐up (months)**: 12
Jennings 2014 24	**Location**: NHS Fakenham weight management service, England **Design**: Cohort study **Period of the study**: 2011–2012 **Recruitment**: Referrals were accepted from General Practitioners **Number of participants allocated**: 230	**Inclusion criteria**: Age >18 years with a BMI of ≥40 kg m^−2^ or ≥ 35 kg m^−2^ with associated comorbidities and/or waist circumference ≥102 cm in men or ≥88 cm in women **Exclusion criteria**: pregnancy, severe eating disorder, poor motivation identified by a motivational questionnaire, or failure to respond to an invitation to contact the service **Baseline age, mean (SD)**: 52.7 (13.6) **Comorbidities at baseline**: 31.7% had diabetes, 0.43% had impaired fasting glycaemia, 11.7% had ischaemic heart disease, 38.3% had hypertension, 11.7% had sleep apnoea and 31.3% had depression	**Delivered by**: General practitioner with additional training as a bariatric physician, specialist nurses, dietitian, psychological therapist, exercise professional, health trainer and supported by a consultant endocrinologist and public health consultant **Description**: The service aimed to deliver interventions including medical assessment, motivational interviewing to support behaviour change, dietary and activity advice, psychological therapies, drug therapy with orlistat, medically supervised LEDs and assessment for suitability for bariatric surgery. The exercise professional provided both individual and small group sessions at the on‐site gym, and there was a 12‐week exercise referral scheme using local gyms. The number of visits ranged from 10–15 visits for the 1‐year programme **Duration of active intervention**: 12 months **Length of follow‐up (months)**: 12
Ryan 2017 35	**Location**: NHS Specialist weight management service in the North East of England **Design**: Retrospective study **Period of the study**: 2013–2014 **Recruitment**: Participants were referred by General practitioners **Number of participants allocated**: 167	**Inclusion criteria**: BMI of ≥40 kg m^−2^ or ≥ 35 kg m^−2^ with associated comorbidities, registered with a local GP; aged >16 years; with an ability to take charge of their dietary intake; assessed as ‘ready to change’; and have had previous attempts at weight loss **Exclusion criteria**: suspected or diagnosed malignancy, pregnant, or requiring post‐bariatric care (unless previously known to the service) **Baseline age, mean (SD)**: 52.2 (11.9) **Comorbidities at baseline**: Not reported	**Delivered by**: Dietician, physiotherapist, psychologist, metabolic physician/endocrinologist, GP with a specialist interest in obesity management **Description**: In phase 1, patents initially received an individual care plan that included an exercise and physical activity plan; outcomes expected; target weight; behavioural goals; and other tools and educational materials. In phase 2, patients move into group services and treatment according to their specific needs and care plan. In phase 3, patients were discharged from the service with details of the patient's outcomes and an ongoing care plan sent to their GP **Duration of active intervention**: 12 months **Length of follow‐up (months)**: 12
Steele 2017 36 Aintree LOSS	**Location**: Hospital clinic, General Practice (GP) surgeries, community centres and a sports centre in Liverpool, England **Design**: Retrospective study **Period of the study**: 2009–2013 **Recruitment**: Based primarily in the community, and referrals are predominantly received from primary care teams, although referrals are also accepted from elsewhere, including secondary care and community dietetics **Number of participants allocated**: 2457	**Inclusion criteria**: BMI of ≥40 kg m^−2^ or ≥ 35 kg m^−2^ with associated comorbidities **Exclusion criteria**: Not reported **Baseline age, mean (SD)**: 48.6 (13.8) **Comorbidities at baseline**: 26% had diabetes, 21.7% had sleep apnoea, 47.7% had depression, 39.8% had hypertension, 32.4% had hyperlipidaemia, 5.2% had myocardial infarction, 6.8% had ischaemic heat disease, 3.3% had stroke and 47.3 had join pain	**Delivered by**: General practitioners, physician with a special interest in obesity, dieticians and physiotherapists psychologists and occupational therapists **Description**: A personalised management plan agreed from a list of dietetics, physiotherapy, occupational therapy and cognitive analytical and behavioural therapy, as well as group sessions (joint physiotherapy, dietetics and hydrotherapy). Group sessions run for 2 h per week for 12 weeks. Individual reviews took place every 1 to 3 months depending on the intensity of intervention required. Contact with leisure services via swimming session was offered. Orlistat was offered as an option to participants **Duration of active intervention**: 24 months **Length of follow‐up (months)**: 24
Logue 2014 27	**Location**: NHS, Glasgow and Clyde Weight Management Service, Scotland **Design**: Prospective observational study **Period of the study**: 2008–2011 **Recruitment**: Referred by their GP or hospital doctor **Number of participants allocated**: 1838	**Inclusion criteria**: Aged ≥18 years with a BMI of ≥35 kg m^−2^ or ≥30 kg m^−2^ with associated comorbidities **Exclusion criteria**: Not reported **Baseline age, mean (SD)**: 49.1 (13.5) **Comorbidities at baseline**: Not reported	**Delivered by**: Service lead, team leaders, dieticians, clinical psychologists, psychology assistant, physiotherapists, administrative staff and technical support staff **Description**: Educational lifestyle programme that included cognitive behavioural therapy and 600 kcal day^−1^ deficit diet and physical activity advice. Phase 1 comprised nine fortnightly 90 min sessions over a 16 weeks. Then patients could choose to enter phase 2 (three 1 h sessions delivered at monthly intervals plus a range of treatment options including further lifestyle advice, prescribed low calorie diet or orlistat). At the end of phase 2, or directly from the end of phase 1, patients could enter a weight maintenance programme (3rd phase) comprising twelve monthly 1 h sessions. Patients who fail to achieve their target weight loss could choose to repeat phase 2 once more and then enter the maintenance programme or opt for bariatric surgery **Duration of active intervention**: 12 months **Length of follow‐up (months)**: 12
Wallace 2015 37 The ‘Live Life Better’ Service	**Location**: NHS weight management service from Derbyshire County, England **Design**: Cohort study **Period of the study**: 2010–2013 **Recruitment**: Referred by their GP or hospital doctor **Number of participants allocated**: 551	**Inclusion criteria**: BMI of ≥40 kg m^−2^ or ≥ 35 kg m^−2^ with associated comorbidities **Exclusion criteria**: Not reported **Baseline age, mean (SD)**: 45.7 (13.3) **Comorbidities at baseline**: 33.2% had hypertension, 3.8% had ischaemic heart disease, 22.1% had diabetes, 1.1% had stroke, 16.3% had asthma, 24.9% chronic join problems, 11.8% osteoarthritis	**Delivered by**: Psychologist, dietitian or physiotherapist **Description**: An intensive lifestyle modification‐based programme involving psychological support, behaviour change strategies, physical activity, dietetic advice and occupational therapy where relevant. (No further details are provided) **Duration of active intervention**: 24 months **Length of follow‐up (months)**: 24
Commercial programmes			
Rolland 2014 34 LighterLife Total	**Location**: Scotland **Design**: Retrospective study **Period of the study**: 2007–2010 **Recruitment**: Self‐referred **Number of participants allocated**: 5965	**Inclusion criteria**: ≥ 30 kg m^−2^ **Exclusion criteria**: Type 1 diabetes, porphyria, lactose intolerance, major cardiovascular or cerebrovascular disease, history of renal disorder or hepatic disease, cancer; epilepsy, major depressive major psychiatric or eating disorders, pregnant or breastfeeding, have given birth or had a miscarriage in the last 3 months **Baseline age, mean (SD)**: 45.6 (10.2) **Comorbidities at baseline**: Not reported	**Delivered by**: ‘Trained weight management counsellors’ **Description**: LighterLife Total VLED programme (550 kcal day^−1^) and group support (in small, single‐sex, weekly groups for the facilitation of behaviour change for the treatment of obesity), along with behavioural therapy. **Duration of active intervention**: Not reported **Length of follow‐up (months)**: 12

GP, general practitoner; LED, low‐energy formula diet (800–1200 kcal day^−1^); VLED, very‐low‐energy formula diet (<800 kcal day^−1^).
